# Toward a Protocol for Transmasculine Voice: A Service Evaluation of the Voice and Communication Therapy Group Program, Including Long-Term Follow-Up for Trans Men at the London Gender Identity Clinic

**DOI:** 10.1089/trgh.2019.0011

**Published:** 2019-05-16

**Authors:** Matthew Mills, Gillie Stoneham, Skye Davies

**Affiliations:** London Gender Identity Clinic, Tavistock and Portman NHS Foundation Trust, London, United Kingdom.

**Keywords:** communication, group therapy, transmasculine, vocal function, vocal situation, voice

## Abstract

**Purpose:** A service evaluation was undertaken with 10 participants identifying as trans men who received voice and communication group therapy and 12-month follow-up at the London Gender Identity Clinic between February 2017 and March 2018, to investigate levels of satisfaction, how helpful they found the program in facilitating vocal change and skill development, and whether they would recommend it to others.

**Methods:** Participant evaluations of *overall* and *ideal* rating of masculinity of voice, and level of feeling *comfortable* with voice, evaluations of voice skills and changes in speaking and reading fundamental frequency were retrospectively reviewed and analyzed.

**Results:** Six participants reported being *very satisfied* with the service; four were *satisfied*. Eight participants found the program *very helpful* in achieving voice and communication change; two found it *helpful*. Eight *strongly agreed* and two *agreed* with recommending the service. Participants' overall and comfort ratings of voice significantly increased (*p*<0.01), while there was no significant change in ideal ratings (*p*=0.063), and a significant decrease in the difference between overall and ideal ratings (*p*<0.01). Participants achieved a significant decrease in fundamental frequency for reading and speaking (*p*<0.01), a significant decrease in voice fatigue (*p*=0.039) and restriction in voice adaptability (*p*<0.01), a significant increase in confidence in public speaking (*p*<0.01), but no significant change in vocal projection (*p*=0.07).

**Conclusion:** Ten trans men reported high levels of satisfaction with the voice group program and long-term follow-up, making significant positive shifts in voice skills and vocal self-perception. These findings apply locally but suggest appropriate interventions toward a transmasculine voice modification protocol.

## Introduction

Transmasculine people form a diverse group,^1^ and studies addressing the invisibility of this population, the psychosocial impact of voice, self-perception of vocal masculinity, and experience of voice and communication therapy services are starting to emerge.^2,3^ “Transmasculine” is an overarching term used in this article to refer to individuals assigned female at birth who have a more masculine, sometimes nonbinary, identity; signaling birth assignation, though, requires sensitive handling as it may be experienced as shaming.^4,5^ Vocal researchers tend to report lowering of the speaking fundamental frequency (F_0_) as a result of the action of exogenous androgen therapy in thickening vocal fold mass to gender-confirming and satisfactorily masculine-sounding levels.^2,3,6–8^ This has led to claims that transmasculine people experience fewer barriers to achieving their desired vocal identity than transfeminine people,^9,10^ and that transmasculine voice therapy is unnecessary.^11^

However, while self-perception of voice improves for many transmasculine people,^12^ pitch change outcomes with testosterone can be highly variable^13,14^ and satisfaction levels with vocal change suboptimal.^15^ Davies et al.^16^ emphasize that transmasculine individuals commenced on testosterone frequently report an enduring difference between their habitual and “passing” pitch and a high occurrence of vocal misgendering. Indeed, there is growing evidence that transmasculine people have particular needs beyond the testosterone-induced effect on pitch in terms of developing voice and communication skills in dynamic psychosocial contexts.^14,17^ Azul^1^ considers the “vocal situation” of transmasculine speakers to be potentially challenging as a result of the interplay between complex factors: presentational (the anatomy and physiology of the speaker/singer's voice and their vocal-communicative behaviors), attributional (the listener's perception and meanings attributed to the speaker/singer's voice), and normative (the cultural, environmental, and heterocisnormative lens through which concepts of gendered voice and vocal function are viewed and experienced).

Clinical practice needs to take into account the diversity of this population and the complexity of factors influencing successful and effective voice function as part of gender congruence.^18^ Nygren et al.^19^ recommend systematic assessment and a therapy focus, addressing safe vocal change as part of complete identity. Transmasculine people's vocal identities are beginning to be understood within and beyond a desire to achieve an unequivocal masculine end-result in binary cisnormative terms.^20,21^ Tackling the social invisibility of this population and creating opportunities for transmasculine people to discover personal vocal and communicative authenticity are paramount.^15,16,22^

Voice and communication interventions applied to transmasculine people are beginning to be tested.^16,21,22^ Azul et al.^18^ state that more research is needed exploring the parameters of functional voice production and communication skills relevant to transmasculine people, placing participants' self-evaluation at the forefront of the enquiry. Mills et al.^21^ report early stages of developing a voice and communication protocol for transmasculine people through a pilot and follow-up voice group program.

Azul's factors^1^ above formed a useful framework for program content that addressed vocal dynamics^21–23^ as part of presence and personal impact,^24^ and took account of the perception of others (Table 1). Client input on what was considered most useful in voice therapy was central to producing a practical guide for trans and nonbinary people, including transmasculine voice.^17^ In addition, studies using approaches that are solution focused,^25^ mindful,^26^ systematic,^27^ narrative,^28^ and compassion focused^29^ further informed interventions in vocal dynamics (pitch, resonance, loudness, intonation, voice quality)^17,23,30,31^ and social communication (public speaking, projection, assertiveness, nonverbal signals, presence)^17,21,22,24^ offered in group contexts.^16,17,21,32^ Group therapy programs have been reported as effective for transfeminine and transmasculine people because group cohesion, commonality of experience, shared learning, feedback, and witnessing, all act as a catalyst for voice and communication change.^17,21,22,32^

**Table 1.** Summary of the Voice and Communication Therapy Interventions Used During the Group Sessions

	Attributional factors	Presentational factors	Diversity	Normative factors
	Self-perception of voice regarding gender Gender attribution by others	Methods used to change gender presentation Anatomical dimensions of voice organ Gender-related voice features	Subject position regarding gender Methods used to change gender presentation	Standards of masculinity and femininity
Voice and communication therapy intervention and focus	Perceptual ratings of overall, ideal, and comfort voice^22^	Vocal embodiment: effects of binding, rib and back stretches^17,21–23,31^	Posture and embodiment of voice^17,22,31^	Laryngograph pitch measurement and discussion regarding cisnormative parameters^16,17,21,22^
	Managing risks of speaking up^17,21,22^	Exploring safe pitch change with or without testosterone^17,21,22^	Presence and personal impact^24^	
	Feedback and discussion from group members^17^	Resonance: jaw and base of tongue release^17,21,22,27,31^	Mindfulness^17,26^	Presence and personal impact^24^
	Follow-up and review sessions^17,21,22^	Optimizing breath support with increased vocal fold mass from testosterone^17,22,30,31^	Compassion focused awareness^29^	Group discussion of authenticity, heterocisnormative bias, and stereotyping^17,20–22^
		Resonance: developing chest and pharyngeal resonance with low humming, chest tapping, yawn talk^17,21–23,31^	Group process and trust and collaboration^15^	Assertiveness training^17,21,22,24^
		Voice education and voice care: managing changes on testosterone and optimizing efficient power-source relationship^17,20,22^	Role play scenarios and improvisation^17,21,22^	Role play scenarios and improvisation^17,21,22^
		Interrelation between loudness and intonation parameters^17,21,22^		
		Voice projection and articulatory muscularity^17,21–23,30,31^		

This article describes a service evaluation of the voice and communication therapy group program at the London Gender Identity Clinic, which consisted of two workshops, and follow-ups at 6 and 12-months for a group identifying specifically as trans men (a subgroup of transmasculine people identifying as men while affirming their history as assigned female sex at birth). The aims were to investigate levels of service user satisfaction, how helpful they found the program in facilitating vocal change and skill development (indicated by self-perception ratings and pitch measures), and whether they would recommend the program to other service users.

## Methods

### Design

The service evaluation received written approval from the Tavistock and Portman NHS Foundation Trust Clinical Audit Offices, and service users' informed consent for participation in the evaluation was gathered before the start of the project. It involved a retrospective review of clinical data of one cohort of voice group participants between February 2017 and March 2018, which included qualitative service evaluation questionnaires, participant self-evaluations of voice and voice skills, and follow-up interview, and quantitative measures of modal speaking and reading fundamental frequency (SFF and RFF). It describes a sample of 10 transmasculine people, identifying as trans men, who attended a generic information-giving seminar as a waiting list initiative and subsequently participated in the voice masculinization therapy group program. This program, delivered by two senior gender specialist speech and language therapists, consisted of two 3-h workshops held a month apart, with follow-up appointments at 6 months, and then, the 12-month post-workshop 2. Workshops took account of Azul's “vocal situation” framework,^1^ delivering interventions in voice change mechanics and communication, shown in Table 1.

Participants' mean age was 26.2 years (range 19–43 years). Five participants had commenced testosterone before the workshop but reported dissatisfaction with their vocal development. Of these five participants, mean length of time on testosterone was 11.6 months (range 6–18 months). Four other participants commenced testosterone at the 6-month follow-up, and one stopped before the 12-month follow-up; one participant preferred not to start testosterone at all.

### Measures

Participants filled out a service evaluation questionnaire where they were asked how satisfied they were with the voice group (1=very dissatisfied, 5=very satisfied), how helpful they found the program (1=very unhelpful, 5=very helpful), and the extent to which they agreed with a statement about recommending the service to others (1=strongly disagree, 5=strongly agree). Participants also filled out a self-report questionnaire in which they were asked to rate their voice on three overarching dimensions: the overall perception of how their voice sounded on a feminine-to-masculine scale (1=very feminine, 10=very masculine), how they would ideally like their voice to sound (using the same scale), and how comfortable they felt with their voice (1=very uncomfortable, 10=very comfortable). Measures were taken at the beginning of the first workshop, at the end of the second workshop, and at the 6- and 12-month follow-up time points.

Participants were also asked to rate their development in a number of voice and communication skills at the beginning of workshop 1 and the end of workshop 2: vocal adaptability, voice projection, public speaking and relational presence, and vocal stamina (reduction in vocal fatigue). Objective laryngographic pitch measures of RFF and SFF were taken at the beginning of workshop 1 and the end of workshop 2. *The Rainbow Passage*^33^ was used for a reading sample and a 2-min monologue topic on a hobby/interest was used for speaking. Brief focused interviews were conducted at 12-month follow-up, in which participants completed service evaluation questionnaires and were asked to relate what had been significant in their voice and communication journey in terms of skills and progress.

All data were reviewed by the two senior treating speech and language therapists, verified by a third senior speech and language therapist in the service, and analyzed by an assistant psychologist and researcher. Variables were identified as attendance rates, motivation with exploration and home practice, timing of testosterone therapy, and participant experience of vocal change process.

### Statistical and thematic analysis

Four-level repeated-measures analysis of variance (ANOVA) and paired sample *t*-tests were conducted to identify any significant changes in the measures (participant evaluations and pitch measures) across the different time points. Participant interview narratives at the 12-month follow-up were reviewed. Raw data were coded for theme development and interpretation based on theme frequency, juxtaposition, interrelationship of participant meaning-making, and experience of voice group and voice and communication development process.

## Results

### Service evaluation

**FIG. 1.**
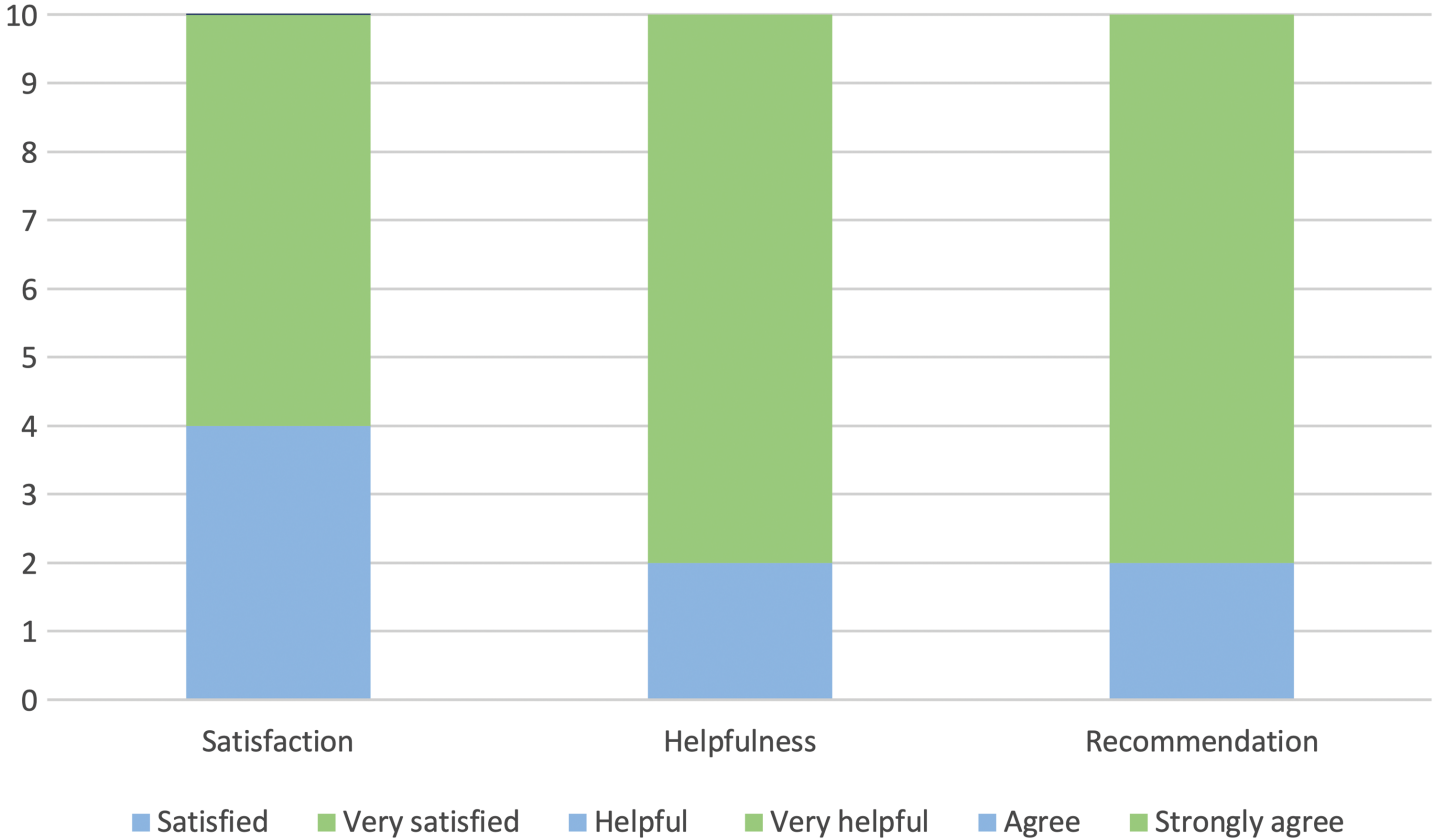
Proportion of responses to service evaluation questionnaires. The figure shows the satisfaction, helpfulness, and recommendation service evaluation responses.

When asked how satisfied they were with the voice group, four participants said they were satisfied and six said they were very satisfied. When asked how helpful they found the group, two participants said they found it helpful and eight said they found it very helpful. When asked if they would recommend the service to others, two participants said they agreed and eight said that they strongly agreed. These results are shown in Figure 1.

### Participant self-evaluations

**FIG. 2.**
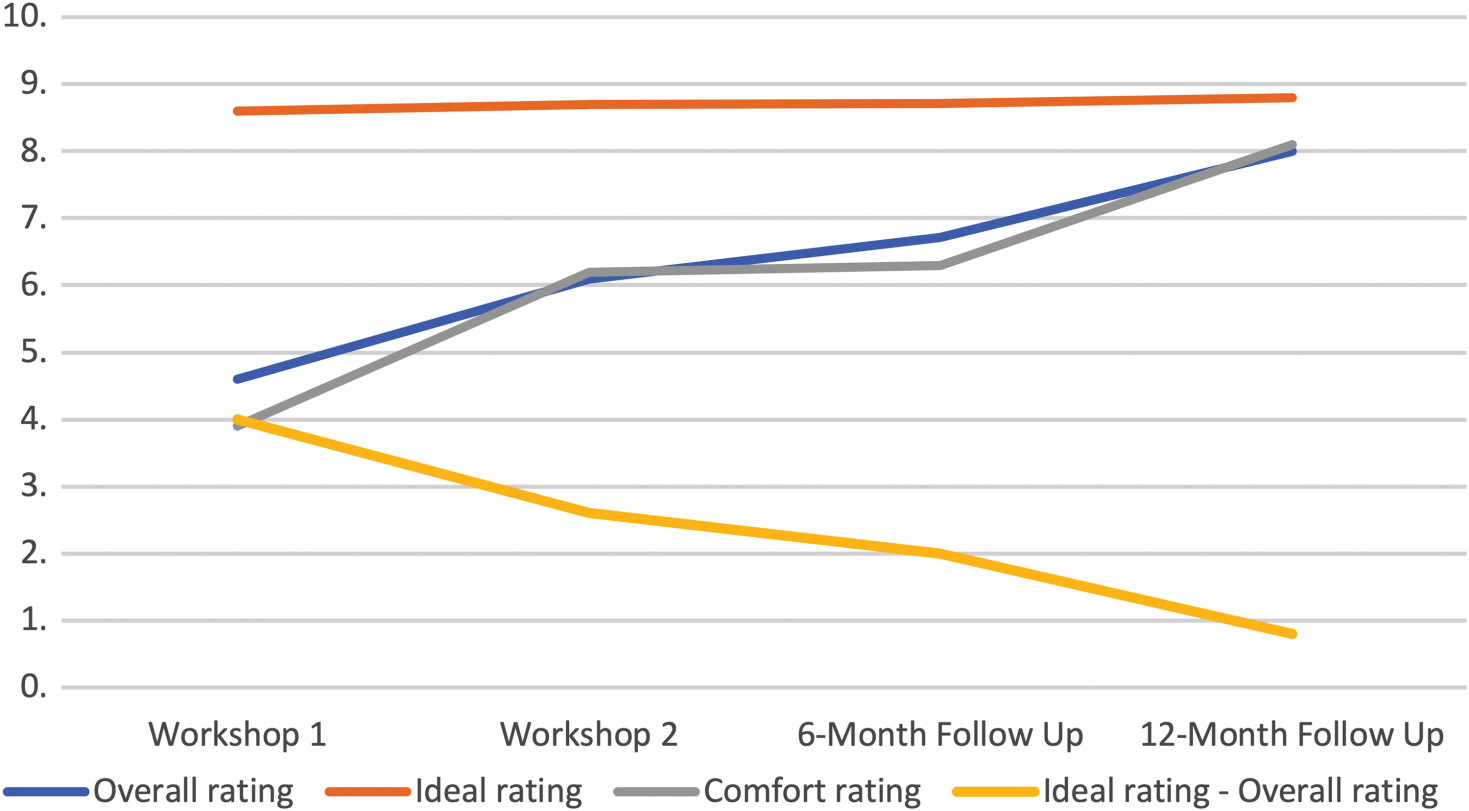
Mean scores for overall, ideal, and comfort ratings across the four time points. The figure shows the changes in mean overall, ideal, and comfort ratings over the four time points (workshop 1, workshop 2, 6-month follow-up and 12-month follow-up).

Figure 2 shows the changes in mean overall, ideal, and comfort ratings over the four time points, as well as the difference between the overall and ideal ratings. Mean scores and standard deviations for responses to the main questionnaire are shown in Table 2. To measure the changes in overall, ideal, and comfort ratings across the four time slots, a four-level repeated-measures ANOVA was conducted for each of the three measures. When an analysis indicated a statistically significant change across time slots, a series of paired sample *t*-tests were conducted between each time point to identify which differences were significant.

**Table 2.** Means and Standard Deviations for Overall, Comfort, and Ideal Ratings and for the Difference Between Overall and Ideal Ratings

	Workshop 1	Workshop 2	6-month follow-up	12-month follow-up
Overall rating	*M*=4.6	*M*=6.1	*M*=6.71	*M*=8
	*SD*=1.71	*SD =* 1.6	*SD =* 1.39	*SD*=1.54
Ideal rating	*M =* 8.6	*M =* 8.7	*M =* 8.71	*M =* 8.8
	*SD*=0.97	*SD =* 0.95	*SD*=0.91	*SD =* 1.03
Comfort rating	*M =* 3.9	*M =* 6.2	*M =* 6.29	*M =* 8.1
	*SD =* 1.2	*SD =* 1.32	*SD =* 0.91	*SD =* 0.74
The difference between overall and ideal ratings	*M =* 4	*M =* 2.6	*M =* 2	*M =* 0.8
	*SD =* 1.49	*SD =* 1.17	*SD =* 1.05	*SD =* 1.03

M, mean; SD, standard deviation.

#### Overall rating

A repeated-measures ANOVA showed a significant increase in overall ratings across the four time points [*F*(3, 27)=45.39, *p*<0.01, η^2^=0.43]. Six paired sample *t*-tests demonstrated significant increases over time between each pairing of time points, as shown in Table 3.

**Table 3.** The Results of Six Paired Sample *t*-Tests for Overall Rating Between the Four Different Time Slots

	Workshop 2	6-month follow-up	12-month follow-up
Workshop 1	*t*(9)=5.58, *p*<0.01, *d =* 1.77	*t*(9)=6.79, *p*<0.01, *d =* 2.15	*t*(9)=9.16, *p*<0.01, *d =* 2.9
Workshop 2		*t*(9)=2.62, *p*=0.02, *d =* 0.83	*t*(9)=6.04, *p*<0.01, *d =* 1.91
6-month follow-up			*t*(9)=4.98, *p*<0.01, *d =* 1.58

#### Ideal voice rating

A repeated-measures ANOVA showed that there was no significant difference for the ideal voice rating across the four time points [*F*(3, 27)=0.59, *p*=0.63, η^2^=0.006].

#### Comfort rating

A repeated-measures ANOVA showed that there was a significant increase between the comfort ratings across the four time points [*F*(3, 39)=47.83, *p*<0.01, η^2^=0.68]. Six paired sample *t*-tests demonstrated significant increases over time between each pairing of time slots, except between the second workshop and the 6-month follow-up (Table 4).

**Table 4.** The Results of Six Paired Sample *t*-Tests for Comfort Rating Between the Four Different Time Slots

	Workshop 2	6-month follow-up	12-month follow-up
Workshop 1	*t*(9)=5.13, *p*<0.01, *d =* 1.62	*t*(9)=9.66, *p*<0.01, *d =* 3.06	*t*(9)=10.09, *p*<0.01, *d =* 3.19
Workshop 2		*t*(9)=0.27, *p*=0.79, *d =* 0.09	*t*(9)=5.46, *p*<0.01, *d =* 1.73
6-month follow-up			*t*(9)=6.18, *p*<0.01, *d =* 1.96

#### The difference between overall and ideal ratings

To assess how the difference between the overall and ideal ratings changed over time, a repeated-measures ANOVA was conducted. The results of the analysis showed a significant decrease in difference between the two across the four time slots [*F*(3, 27)=26.7, *p*<0.01, η^2^=0.51]. Six paired sample *t*-tests demonstrated significant decreases over time between each pairing of time slots, except between the second workshop and the 6-month follow-up (Table 5).

**Table 5.** The Results of Six Paired Sample *t*-Tests for the Difference Between Overall and Ideal Ratings Across the Four Different Time Slots

	Workshop 2	6-month follow-up	12-month follow-up
Workshop 1	*t*(9)=4.12, *p*=0.003, *d*=1.05	*t*(9)=5.07, *p*<0.01, *d*=1.56	*t*(9)=7.24, *p*<0.01, *d =* 2.51
Workshop 2		*t*(9)=1.77, *p*=0.111, *d =* 0.54	*t*(9)=5.01, *p*<0.01, *d*=1.63
6-month follow-up			*t*(9)=4.13, *p*=0.001, *d =* 1.15

### Voice and communication skills

**FIG. 3.**
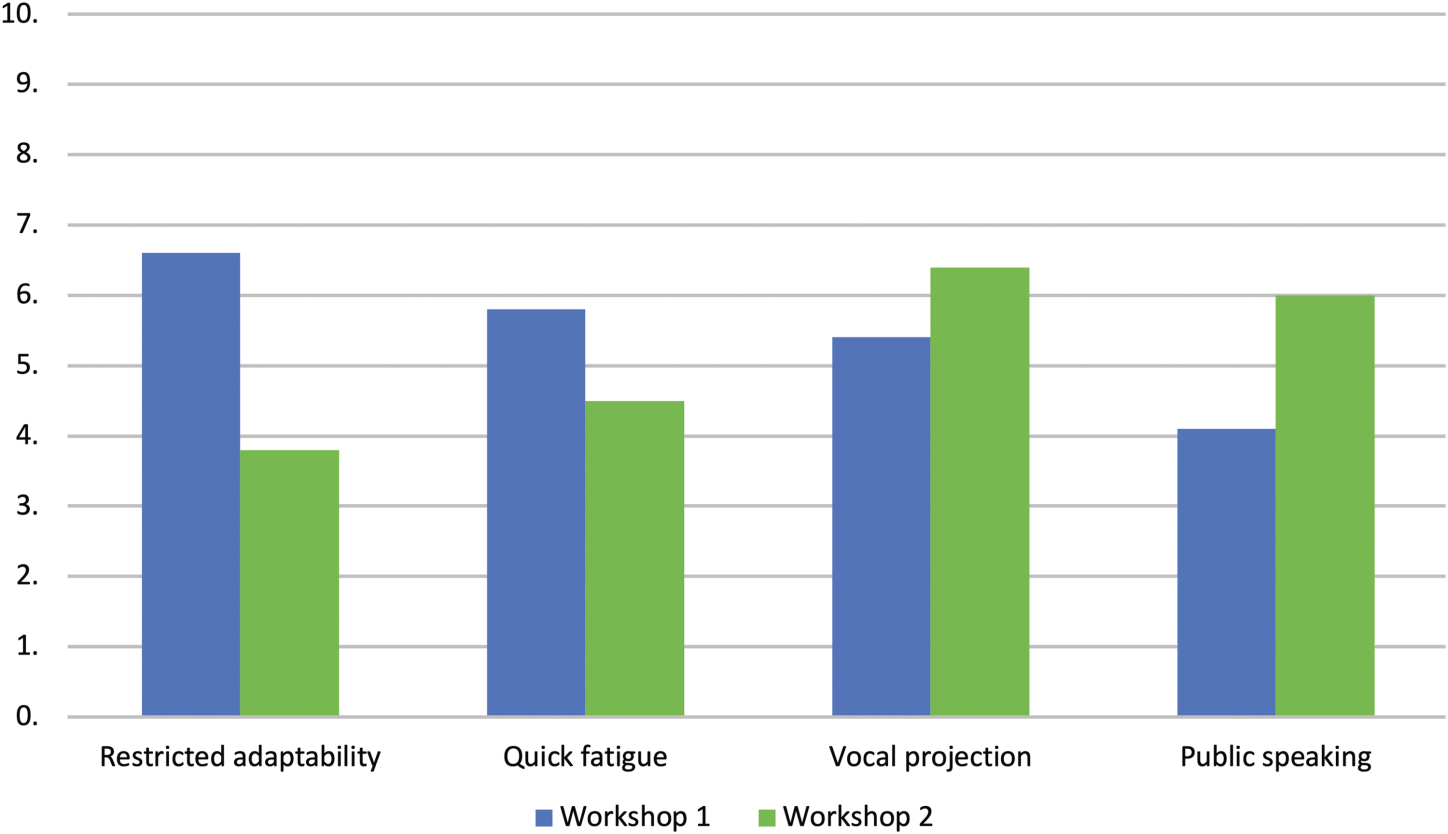
Mean scores for participants' rating of restricted voice adaptability, quick fatigue, vocal projection, and confidence in public speaking. The figure shows the differences in mean ratings for vocal skill measures.

At the beginning of the first and at the end of the second workshops, participants were asked to evaluate their skills in voice and communication—specifically: the extent to which the adaptability of their voice was restricted, how quickly their voice would fatigue, their ability to project their voice, and their confidence in public speaking. Figure 3 shows the differences in mean ratings for these measures. Means and standard deviations are shown in Table 6.

**Table 6.** Means and Standard Deviations for Participants' Ratings of Vocal Skills

	Workshop 1	Workshop 2
Restricted adaptability	*M =* 6.6	*M =* 3.8
	*SD =* 1.58	*SD =* 1.48
Quick fatigue	*M =* 5.8	*M =* 4.5
	*SD =* 2.9	*SD =* 2.37
Vocal projection	*M =* 5.4	*M =* 6.4
	*SD =* 2.27	*SD =* 1.65
Public speaking	*M =* 4.1	*M =* 6
	*SD =* 2.23	*SD =* 1.94

To see if there was a significant change in self-reported levels of vocal skills between the two workshops, four paired sample *t-*tests were conducted comparing each of the four items at the first workshop and the second workshop. The analyses showed a significant decrease in ratings of vocal fatigue [*t*(9)=2.41, *p*=0.039, *d*=0.76] and restriction on the adaptability of voice [*t*(9)=6, *p*<0.01, *d*=1.9], and a significant increase in confidence in public speaking [*t*(9)=5.02, *p*<0.01, *d*=1.59]. There was an increase in reported levels of vocal projection between the first and second workshops, however, this increase was nonsignificant [*t*(9)=2.02, *p*=0.07, *d*=0.64].

### Pitch measures

At the beginning of the first and at the end of the second workshops, participants' modal RFF and SFF pitches were measured. Means and standard deviations for speaking and reading pitch are shown in Table 7. To see if there was a significant change in pitch between the two workshops, two paired samples *t*-tests were conducted comparing the reading and speaking levels of pitch between the first and second workshops. These tests showed significant decreases for both speaking pitch [*t*(9)=4.47, *p*<0.01, *d*=1.41] and reading pitch [*t*(9)=4.37, *p*<0.01, *d*=1.38] between the two workshops.

**Table 7.** Means and Standard Deviations for Reading and Speaking Modal Pitch Between the Two Workshops

	Workshop 1	Workshop 2
Speaking pitch	*M*=161	*M*=143.2
	*SD*=34.08	*SD*=30.3
Reading pitch	*M*=165.7	*M*=154.1
	*SD*=35.73	*SD*=29.87

### Qualitative thematic analysis

Participant clinical interviews at the 12-month follow-up were coded, and a thematic analysis undertaken in terms of frequency of key words, phrases and common narratives of perceptions, feelings and experiences of voice and communication therapy, vocal function and development, and being in the group.

#### Themes

*Group learning:* “voice group and review really helped me to learn about how to use my voice better and more effectively at work and on the phone”; “the group was super important as a safe space to explore not just my voice but communication.”

*Embodying voice:* “learning voice projection and being assertive, and linking up my voice to my body has helped me with public speaking”; “my voice is hooked up to my body more now.”

*Managing challenge and developing confidence:* “I can be more assertive in meetings now”; “I have been able to raise the bar higher as I have developed my voice more.”

*Voice exploration beyond pitch change:* “voice therapy was somewhere for my voice to grow in before I started t–that was really surprising and helpful”; “I learned about the difference between loudness and expression in my voice and that was key to my confidence, even though I had already started t.”

## Discussion

The diversity reported by Azul^1^ in the transmasculine population applies to the subpopulation in this sample of individuals who identify as trans men, evidenced by a range of highly personal self-constructs of gendered voice beyond the parameter of pitch alone. Voice and communication group therapy can offer a space not only to explore safe voice change but also those presentational, attributional normative, and diversity factors that contribute to individual style and behavior in social interaction. Notably, all participants were measured to use a personally meaningful and attainable lowered speaking and reading pitch after workshop 2, with no dysphonia (voice disorder). For those five not taking testosterone, pitch lowered by 0.5–1.5 semitones. This is an important implication for clinical practice as a marker for the limits of lowering pitch behaviorally without vocal hyperfunction.

As participants' ratings of their overall sense of voice masculinity and comfort increased significantly from the first workshop to the 12-month follow-up, it seems that the benefits of comprehensive group programs can be both sustainable and transferrable into everyday life. In addition, qualitative data from client narratives at the 12-month follow-up, together with the decrease in the difference between participants' overall and ideal ratings for their voice, suggest that positive achievements were linked to increasing confidence, for example: “I have been able to raise the bar higher as I developed my voice more.” Self-evaluations confirmed that group therapy can address broader aspects of vocal function such as vocal stamina and flexibility, and more confident presentation of self, such as in public speaking. Participant ratings of vocal projection did not differ significantly from the beginning of workshop 1 to end of workshop 2, possibly suggesting that this is an advanced vocal skill requiring more opportunities for development. However, at the 12-month follow-up, narrative themes indicated that there was further development in this parameter (*embodying voice* theme).

All participants reported the service as helpful in facilitating vocal change and voice skill development, and that they would recommend the program to other service users. While this evaluation cannot be generalized to other populations, it indicates that the following interventions addressing vocal function and situation were significant catalysts for change for this specific group, and the details add to what has already been described.^16,17,21,22^ The interventions involved coaching and motor learning of specific voice skills, raising mindful awareness of the felt sense of body and voice in exercises generalizing to discursive contexts, and the choices available to individuals regarding relational, social presence:

- Voice education (vocal anatomy and physiology)^16,17,21,22,32^
- Voice care, in particular during vocal fold changes on testosterone and pitch monitoring^17,21,22^
- Vocal embodiment—effect of binding on resonance, rib and back stretches, jaw release, centered breathing, and grounding^17,21–23,31^
- Optimizing breath support especially regarding vocal mass changes on testosterone^17,22,31^
- Chest and pharyngeal resonance development—chest tapping, low humming, tongue root release, and jaw release^17,22,23,30,31^
- Presence and personal impact^17,21,24^
- Mindfulness and compassion^26,29^
- Role-play and improvisation of everyday speaking situations, for example, telephone speaking and interviewing^17,21,22^
- Voice projection— “twang” voice quality, “arcing” voice, and muscular articulation development^17,21–23,31^
- Assertiveness training^17,21,22^
- Discussion of norms, unconscious bias, and authenticity^17,21,22^
- A focus on solutions and giving/receiving constructive peer feedback.^17,21,22,25^

### Limitations

SFF and RFF pitch measures, and self-evaluations of *voice skill* measures were not taken beyond workshop 2. Repetitions of these measures would have described potential relationship between voice skills and the comfort, overall, and ideal ratings and specific carryover beyond the workshops. Instead, these are thematically suggested in qualitative narrative terms alone. Service evaluation questionnaires were returned anonymously and descriptive statistics only could be described from the sample. The service evaluation findings cannot be generalized to other populations, and the local population sample of 10 is small. The participants, while all identifying as trans men, expressed highly individual voice and communication goals. Therefore, satisfaction with this protocol should be replicated and assessed among larger cohorts, not only of trans men but also of transmasculine and nonbinary individuals seeking masculinizing voice therapy, and in prospective research into the voice and communication therapy interventions.

## Conclusions

Ten trans men receiving voice and communication group therapy and follow-up to 12-months reported high levels of satisfaction with the service, that it was helpful in facilitating voice change and vocal skill development, and that they would recommend it to others. They reported significant shifts in voice skills and self-evaluations of voice. The evaluation demonstrated that voice and communication interventions used in the service are significant in facilitating vocal situational change, and suggest inclusion in the development of a transmasculine voice modification protocol.

## Abbreviations Used

ANOVA: analysis of variance
RFF: reading fundamental frequency
SFF: speaking fundamental frequency
